# Early Detection of Lung Cancer Using Nano-Nose - A Review

**DOI:** 10.2174/1874120701509010228

**Published:** 2015-08-31

**Authors:** M. P. Fernandes, S Venkatesh, B. G Sudarshan

**Affiliations:** 1 Department of Instrumentation Technology and PG Studies, R.V. College of Engineering, Bangalore, India; 2 Department of Instrumentation Technology and PG Studies, R.V. College of Engineering, Bangalore, India; 3 Department of Instrumentation Technology and PG Studies, R.V. College of Engineering, Bangalore, India

**Keywords:** Benchmarks, biomarkers, cancer, chemiresistor, endogenous, exogenous, GC-MS system, IMS, monolayer-capped, phasetransfer, PCA, SPME, TEM, thiol, VOC

## Abstract

Lung cancer is one of the malignancies causing deaths worldwide. The yet to be developed non-invasive diagnostic techniques, are a challenge for early detection of cancer before it progresses to its later stages. The currently available diagnostic methods are expensive or invasive, and are not fit for general screening purposes. Early identification not only helps in detecting primary cancer, but also in treating its secondaries; which creates a need for easily applicable tests to screen individuals at risk. A detailed review of the various screening methods, including the latest trend of breath analysis using gold nanoparticles, to identify cancer at its early stage, are studied here. The VOC based breath biomarkers are used to analyze the exhaled breath of the patients. These biomarkers are utilized by Chemiresistors coated with gold nanoparticles, which are found to be the most suited technique for early detection of lung cancer. This technique is highly accurate and is relatively easy to operate and was tested on smokers and non-smokers. This review also gives as an outline of the fabrication and working of the device Na-Nose. The Chemiresistors coated with Gold nanoparticles, show a great potential in being an non-invasive and cost-effective diagnostic technique for early detection of lung cancer.

## INTRODUCTION

Lung cancer accounts for around 28% of cancer-related deaths worldwide [1]. More than 60% of patients have their symptoms diagnosed at the later stages with longevity of less than 10%. The chances of survival are narrow and at the early stages, it cannot be detected due to the presence of little or no symptoms. Patients diagnosed during their initial phase of lung cancer can live for a period of 5 years [2]. There is a need for a new, simple and innovative technique which is less expensive and non-invasive. Recent years have brought forward a new method for the early diagnosis of Lung cancer by the analysis of the exhaled breath which contains blueprints of gaseous and nongaseous markers that will help in distinguishing the breath of cancer stricken patients from the healthy population [3].

## DETECTION TECHNIQUES

Symptoms like persistent cough, blood filled sputum, pain in the chest, change in the voice pattern and recurrent pneumonia or bronchitis are mainly noticed in the last stage of lung cancer and the techniques used for its detection and diagnosis is expensive. The current techniques used are:

- **Chest X-ray**

Chest X-ray cannot be used in the detection of lung tumours in their earliest stages. Coincidentally around 10% of lung cancer cases are discovered only when people undergo a chest X-ray for other respiratory conditions. Fig. (**1**) shows a chest X-Ray of normal lungs and another chest X-ray pointing to an abnormal mass present inside the lungs.

- **Sputum Cytology**

Sputum contains lung cells, which can be analysed under the microscope using sputum cytology. This test can reveal if the lung cells have fully developed into cancer cells or not.

- **Pulmonary Function Tests (PFT) **

PFT is used to measure the amount of air taken in and released out by the lungs. Since Lung cancer constricts the flow of air in and out of the lungs, it transfers poor amount of oxygen into the blood, thus aiding in lung cancer detection.

- **Chest Tomography**

After the patient is diagnosed by chest X-ray, further examination of lung tissues is done using CT, which gives an indication whether the cancer is present in the lungs or has spread to the other parts of the body.

- **Bronchoscopy with Biopsy**

Examination of the interior region of the lungs can be done using a bronchoscope and a biopsy of a lung nodule or mass taken. This technique can be uncomfortable for one to undergo and the assessment of the biopsied tissue is expensive [3-6].

## HISTORY OF BREATH ANALYSIS

Since ancient times, physicians have known that a person’s breath gives an indication of one’s health conditions. For example, people with diabetes have a ‘fruity’ smell and people with kidney disease have a fish-like smell to their breath [8], which has lead to researchers in discovering its potential as a diagnostic tool. Collection of a breath sample is much safer and easier compared to that of collecting blood samples or urine samples [9, 10] and the compounds present in the breath can be detected and correlated to various diseases. VOCs as illustrated in Fig. (**2**), for lung cancer were first found by Gordon and colleagues in 1985, using Gas Chromatography-Mass Spectrometry (GC-MS) [11].

## BIOMARKERS

National Institute of Health states that a biomarker is an indicator of any normal biological, pathogenic or pharmacological responses that take place in the human body. These biomarkers are present in various regions of the human body such as, the cells of the tumor growth, fluids in the body which include blood, urine, cerebrospinal fluid, sputum, saliva, and breath. Their discovery, will lead to a non-invasive, early detection of a few types of cancer [12]. This has opened up a new beginning for the early diagnosis of cancer and the precise delivery of drugs to the specific cancer regions of the human body [13].

The surface of cancerous tissue emits chemicals in the form of Volatile organic compounds (VOCs) which can be detected by sensors (chemiresistors) on a Nano-sensor array. These sensors can differentiate between the breath of a healthy and cancerous individual. This forms the foundation for a fast, accurate method for detection of cancer [13, 14].

## VOC

The exhaled breath is made up of oxygen, carbon dioxide, nitrogen, water, inert gases and volatile substances. These volatile substances can be produced endogenously or exogenously. The endogenous breath biomarkers for lung cancer are described in Table **1** [15].

Around 42 VOCs represent lung cancer VOC biomarkers, out of which the important VOCs are:

- Acetaldehyde (below 2-10 ppb)
- Formaldehyde (below 2-10 ppb)
- Undecane (24±4 ppb)
- Isopropene (72.9- 81.5 ppb)
- Methanol (below 118.5 ppb)
- Ethylbenzene (145±35 ppb)
- Acetone (below 458.7 ppb)

The Exhaled Breath VOC Analyzers can be further used in the early detection of breast cancer by the detection of VOC biomarkers that include the derivatives of alkanes such as tridecane, hexanal, heptanal, tetradecane and dodecane [16, 17]. Formaldehyde is also found in breast, bladder, and prostate along with 16 other compounds such as toluene; acetic acid; 2,3,4-trimethyl decane; p-xylene; and 2,2-dimethyldecane dodecane; 3,3-dimethyl pentane; 1- iodononane; etc [18-21]. 

## EXHALED BREATH VOC ANALYZERS

The Exhaled breath VOC analyzers are devices used for the detection of the VOCs, whose abnormal levels point to the presence of cancer in the human body. These devices have to be fine-tuned by validating the biomarkers with respect to their pure compounds to achieve high sensitivity, specificity and accuracy for them to be used as a screening tool for the early detection of lung cancer [23]. Following are the types of VOCs analyzers under trial, as screening tool of lung cancer detection.

### GC and Mass Spectrometry (GC-MS) 

GC-MS system is versatile in determining a wide range of VOCs [6]. Gas chromatograph has a high separating efficiency [24] and separates the chemical mixture into pulses of pure chemicals based on their volatility, or ease with which they evaporate into a gas. The MS identifies the VOCs and quantifies them based on their structure [25].

### Electronic Noses 

In the Electronic nose (E-Nose) as portrayed in Fig. (**3**), the VOCs adsorb onto a sensor where a change in conductivity, color or oscillation of a crystal is detected by the sensing system of the E-nose. The E-nose responds to only a mixture of compounds in the sample. The pattern recognition system interprets and detects the high levels of VOCs causing Lung cancer [10, 17, 26-28]. The drawbacks include the extensive preparation of the breath samples, lack of quantitative data and calibration [17, 29].

### Quartz Microbalance 

Quartz microbalance [30] contains piezoelectric sensors. These sensors use highly sensitive quartz crystals for the measurement of the mass and viscosity of the VOCs that interact within its thin films. Molecular interaction between target and ligand is detected by the sensor. The resistance at resonance, changes with the viscosity of the VOCs in contact with the crystal surface, which leads to a frequency shift [31-33]. Difficulty lies in the selection of sensitive polymers for their application as thin films over the sensors and also in the control of the repetition of the coating process [17, 34].

### Colorimetry

The colorimeter [35] distinguishes among the VOCs by its composite response. The Colorimetry sensor array uses a diverse range of chemically responsive dyes.

Adsorption of VOCs causes these dyes to change their colours depending upon their chemical environment which is then scanned and converted to a number [17, 36].

### Ion Mobility Spectrometry (IMS) 

The IMs consists of an ionizing source that breaks the VOCs molecules into ions. The detection process in the IMS, operated in the positive and negative modes, is based on the movement of ions in a drift gas that occurs at the ambient pressure under the influence of stable electric field, in the range from 100 to 350 Vcm^-1, ^towards a Faraday plate [38], thus generating an electrical signal. The electrical signals from each VOC combine to produce an ion spectrum [17, 38, 39].

### Cyranose 320 

Cyranose 320 consists of 32 carbon polymer sensors that respond to exhaled breath, where a change in sensor resistance is caused by the adsorption of the VOC mixtures of the breath onto the sensors. The exhaled breath sample of each individual results in a pattern of sensor responses, which is a characteristic of each individual’s smell print. This helps in distinguishing between the breath of a healthy individual and of a cancer stricken person [39].

The screening techniques described above aren’t cost-effective and are slow. They require the use of complex instruments for the dehumidification of the breath biomarkers. Many of these techniques are difficult to use, thus creating a need for highly experienced analysts for the operation and the interpretation of the results [17]. This creates a need for less complicated breath analysers as shown in Fig. (**4**), that are highly effective for the mass screening of individuals for lung cancer.

## NANO-NOSE

Nano-Nose analyses the gases present in the breath and identifies those gases that stipulate the presence of lung cancer. It works by binding gases to specific chemiresistors coated with gold nanomaterials, thus aiding in the detection of volatile organic compounds (VOC) of the exhaled breath.

## CHEMIRESISTORS USING GOLD NANOPARTICLES

Researchers throughout the world are working on a revolutionary device to detect lung cancer and other types of cancers, using exhaled breaths of the patients. A few scientists have developed a device containing a carbon-based sensor embedded with gold nanoparticles [40].

### Mono-layered Gold Nanoparticles

Gold nanoparticles are highly sensitive in the detection of biomarkers at lower concentration levels [41]. They are fully biocompatible [42] and no scattering occurs when mono-layered gold nanoparticles are used. All interactions with respect to the gold particles occur at the surface. The electron density change in the surface causes a maximum shift in the surface Plasmon absorption, which helps in the detection of VOCs. It thus enables the use of noble metal nanoparticles as sensitive sensors [43].

Mono-layered capped gold nanoparticles are thermally air stable of reduced dispersity, have well controlled size and are well spaced [1, 44]. As the concentration of gold nanoparticles increases, there is an increase in the dc conductivity. When the concentration reaches to a moderate level, the value of the dc conductivity becomes steady. Toluene solvent suspensions and Gold nanoparticle/chloroform are used in the preparation of Mono-layered gold nanoparticles as there is an increase in electrical conductivity due to the presence of shorter molecular chain at the end of the Au nanoparticles [45]. These monolayered Au particles control the properties at interface and have a high surface area-to-volume ratio, which makes them different and much better than the bigger Au molecules. The limit of identification of VOCs by the nanoelectrode is lower than their detection at the macrosized electrode. This is mainly due to the presence of a higher ratio between the faradic and capacitive currents [46]. 

### Chemiresistor

The electrodes present in the Chemiresistors, are coated with a conducting polymer film. The thin electrically- conductive polymer films swell due to the chemical reaction of the volatile organic chemicals with that of the films, thus causing a change in electrical resistance across the film. The chemiresistor can be used repeatedly as it regains its original condition by the release of the chemicals in the environment [48].

## METHODS OF PREPARATION (REVIEW)

### Synthesis of Monolayer-capped Gold Nanoparticles (MCNP)

Metallic nanoparticles are synthesized in a two-phase system which is composed of aqueous and non-aqueous solutions. These solutions are combined with techniques of extraction of ions. The metallic monolayered nanoparticles are then coated with a hydrophobic layer of alkanethiols. First the ions from an aqueous solution are transferred with the help of a phasetransfer reagent to a toluene solution. Then the reduction of gold is done with an aqueous borohydride solution which is later capped with thiols [44]. Lastly the gold nanoparticles are repeatedly extracted and purified from thiols [1]. 

### Sensor Array Patterning and Fabrication

As shown in Fig. (**5**), fourteen different chemiresistive MCNP films deposited on micrometric electrical transducers, are combined onto a sensor array. They are insensitive to the water molecules in the exhaled breath and are placed inside a test chamber [49]. Deposition of 20 circular gold electrodes on thermal oxide silicon wafer is done by the use of an electron beam evaporator. Post-deposition, the gold nanoparticles are then extracted from their chloroform solution by sonication [1].

### Breath Testing

The sensor array is first tested by evacuating the chamber and exposing it to a mixture of artificially designed VOC biomarker breath patterns. The procedure is repeated and the signals obtained, are scrutinized using a standard PCA algorithm [1, 49].

### Principal Component Analysis (PCA)

Analyzing the relationship between multiple variables within a multi-dimensional data, requires a statistical method such as PCA, which can reduce the dimension of the data using linear feature extraction technique. Each sensor data is retained and arranged into a matrix format where the sensor types are arranged in rows and the measurement numbers are arranged in columns. The percentage-resistance changes and slopes are used in the calculation of the covariance matrix and its values of the Eigenvector and Eigen value. The two principle components discriminate between non-overlapping healthy and lung cancer group. This is used in the interpretation of PCA results, in the score plot containing the projection of two cluster data onto the two dimensional planes [40]. The data is then confirmed by testing the breath patterns using Solidphase Microextraction (SPME) and GC-MS.

### A COMPARISON OF EXHALED BREATH ANALYZERS 

The highly sensitive and accurate GC-MS, with 87% reproducibility, are rarely used because they are expensive, difficult to use and require highly experienced analysts to operate them and interpret the results [1, 17]. 

Electronic nose cannot obtain quantitative data as the sensors are not selective. If more than one type of vapour is present, a quantitative output from one sensor is likely to be erroneous [50] and therefore the device cannot be calibrated. 

Quartz microbalance gives an accuracy of 80%, sensitivity of 85% with 100% specificity. Their main drawback lies in the stability and precision due to the requirement for the pre-concentration of the breath samples collected [51, 52].

The sensitivity of the Colorimeter is 73.3% and specificity 72.4%. They are generally less preferred due to their low sensitivity and the inability to work in the presence of humidity and the need for the pre-concentration of the collected breath [17].

The accuracy of Cyranose 360 is 85% but is not used as it’s slow, costly, requires complex instruments and cannot detect VOCs in the presence of humidity [17].

The electronic nose containing chemiresistors coated with gold nanoparticles have an accuracy of around 88%. The long chain thiols used in the synthesis of the monolayered gold particles differ in their polarity and vapor pressure. This provides good stability to the device for a period of three months [54]. Change in the electrical resistance of the film occurs due to the absorption of the breath onto the sensor array. As a result, these chemiresistors can identify the pattern of each breath biomarker and therefore quantitative data can be obtained [51]. Pre-concentration of the breath samples is not required due to the hydrophobic nature of the thiol coating on the Au nanoparticles. It has a greater reproducibility of 90%, which proves that is more accurate, promising and convenient to be used as a method for lung cancer detection at its early stages [1].

The advantages of this technique include a high accuracy in discriminating between malignant and benign disease. The breath analysis test can be repeated in short intervals, without the requirement for dehumidification. Large volume of sample can be collected and the test is easy to perform. It can be tested on smokers as well as non-smokers and has a great potential as a screening test. The limitations of exhaled breath analysis include the lack of recommended guidelines for sampling of exhaled breath. Also a need arises, to bring down the cost of the gold nanoparticles. Collection and analysis of the samples using techniques such as mass spectrometry and infrared spectroscopy also hinder in the faster analysis of the breath biomarkers.

## CONCLUSION

For the early detection of lung cancer, standard diagnostic techniques cannot be used as they detect abnormalities at the later stages. An innovative, portable technique is required for the early detection of lung cancer. VOC biomarker testing in the samples of exhaled breath is a non-invasive approach. Nanotechnology can be used in the development of a simple, portable and effective screening device for lung cancer, as the surface area of the nanoparticles is high, making them more suitable as breath biomarkers. A Chemiresistor uses monolayered gold nanoparticles for being biocompatible and their ability to detect VOCs at very low concentrations. A much greater potential exists if this technology can be used to diagnose other types of cancer, which could mean enhanced opportunities to save lives [1, 53] and [14].

## Figures and Tables

**Fig. (1):**
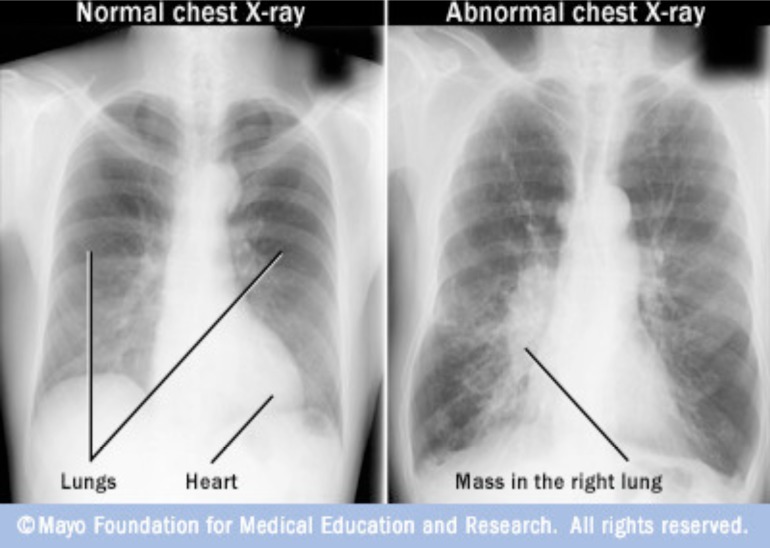
Chest X-Ray of normal lungs and cancer affected lungs [7].

**Fig. (2):**
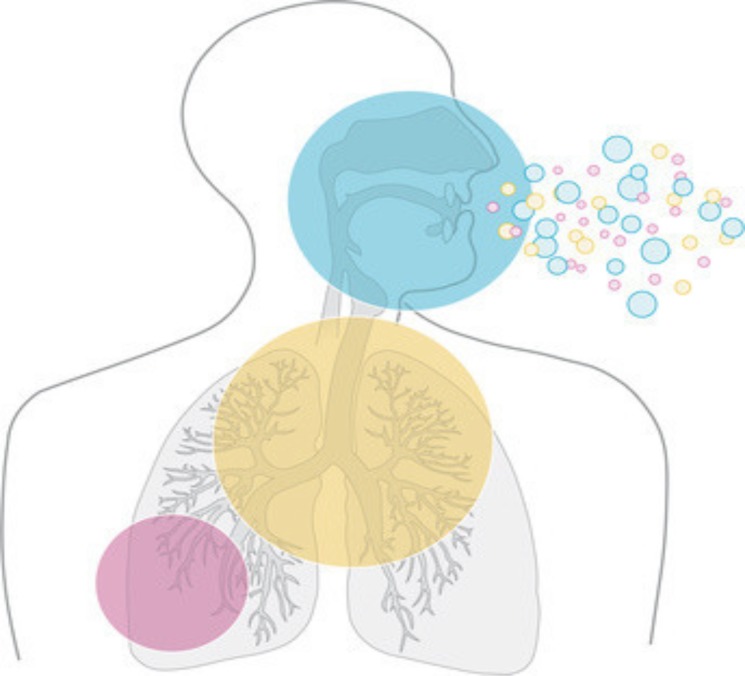
VOC [22].

**Fig. (3):**
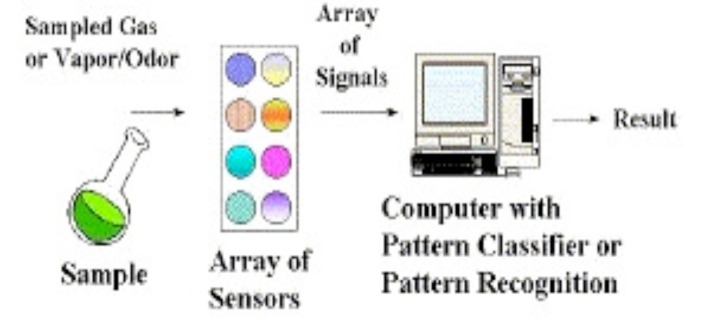
Working of electronic nose [37].

**Fig. (4):**
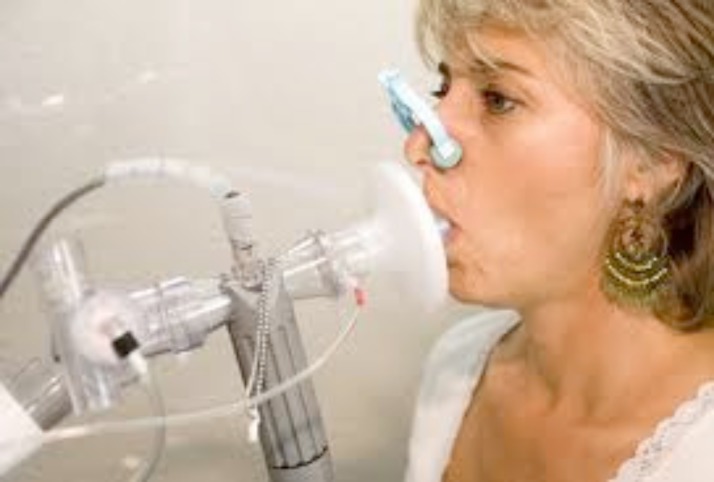
Control using the breath analyzer for the detection of lung cancer [47].

**Fig. (5):**
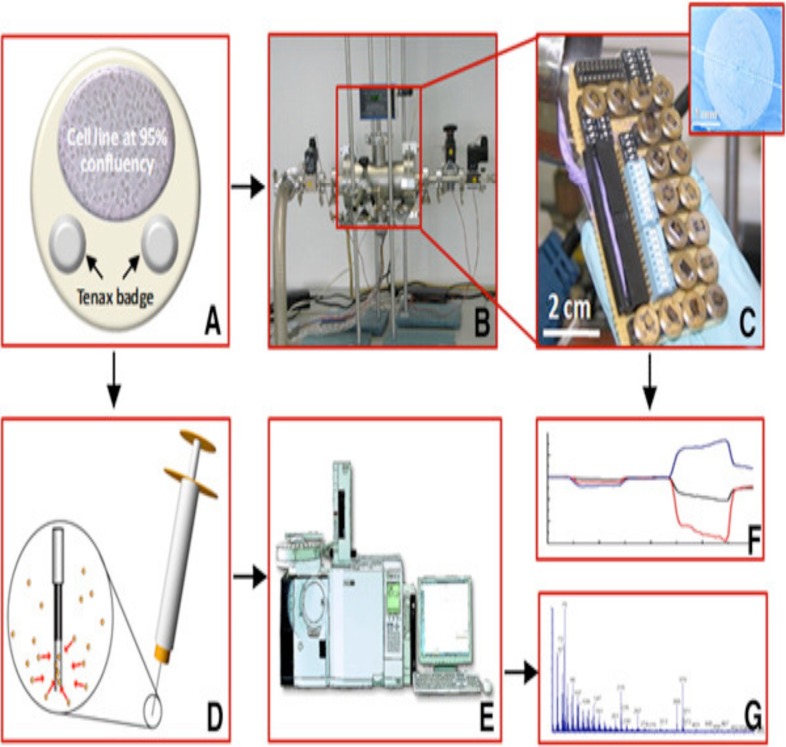
Schematic representation of lung cancer breath testing using sensor array containing monolayered gold nanoparticles. (a) Chemiresistor array, (b) and its SEM image, (c) SEM image of Au nanoparticles on the sensor array (d) with its transmission electron micrograph (TEM) image (e, f, g) electronic testing of breath [1].

**Table 1.** Types of VOCs [36].

Class of VOC	Example	Mechanism of Production
Saturated hydrocarbons	Ethane, aldehydes, pentane	Peroxidation of lipids
Unsaturated hydrocarbons	Isoprene	Synthesis of cholesterol by the Mevalonic pathway
VOCs containing oxygen	Acetone	Decarboxylation of acetoacetate
VOCs containing sulphur	Dimethylsulfide, ethyl mercaptane,	Methionine- Incomplete metabolism
VOCS containing nitrogen	Ammonia, dimethylamine	During elevated levels in the urine

## FUTURE SCOPE

Breath analysis complements blood and imaging techniques is the future of medical testing, making it suitable for the early detection of lung cancer. There are broader applications of this technology wherein the breath biomarkers present in exhaled breath can also be used in the detection of colon cancer, breast cancer, rectal cancer and bladder cancer. The discovery of these revolutionary electronic and chemical sniffers by the scientists will help in the examination of the exhaled breath for signs of other maladies such tuberculosis, asthma and other types of cancer.
